# The SKA3-DUSP2 Axis Promotes Gastric Cancer Tumorigenesis and Epithelial-Mesenchymal Transition by Activating the MAPK/ERK Pathway

**DOI:** 10.3389/fphar.2022.777612

**Published:** 2022-02-28

**Authors:** Chao Zhang, Shutao Zhao, Yuen Tan, Siwei Pan, Wen An, Qingchuan Chen, Xudong Wang, Huimian Xu

**Affiliations:** ^1^ Department of Gastrointestinal Nutrition and Hernia Surgery, The Second Hospital of Jilin University, Changchun, China; ^2^ Department of Surgical Oncology, First Affiliated Hospital of China Medical University, Shenyang, China; ^3^ Key Laboratory of Gastric Cancer Molecular Pathology of Liaoning Province, Shenyang, China

**Keywords:** gastric cancer, SKA3, DUSP2, EMT, MAPK/ERK

## Abstract

**Background:** Spindle and kinetochore-related complex subunit 3 (SKA3), a member of the SKA family of proteins, is associated with the progression of multiple cancers. However, the role of SKA3 in gastric cancer has not been studied.

**Methods:** The expression levels of SKA3 and dual-specificity phosphatase 2 (DUSP2) proteins were detected by immunohistochemistry. The effects of SKA3 and DUSP2 on the proliferation, migration, invasion, adhesion, and epithelial-mesenchymal transition of gastric cancer were studied *in vitro* and *in vivo*.

**Results:** Immunohistochemical analysis of 164 cases of gastric cancer revealed that high expression of SKA3 was negatively correlated with DUSP2 expression and related to N stage, peritoneal metastasis, and poor prognosis. *In vitro* studies showed that silencing SKA3 expression inhibited the proliferation, migration, invasion, adhesion and epithelial-mesenchymal transition of gastric cancer. *In vivo* experiments showed that silencing SKA3 inhibited tumor growth and peritoneal metastasis. Mechanistically, SKA3 negative regulates the tumor suppressor DUSP2 and activates the MAPK/ERK pathway to promote gastric cancer.

**Conclusion:** Our results indicate that the SKA3-DUSP2-ERK1/2 axis is involved in the regulation of gastric cancer progression, and SKA3 is a potential therapeutic target for gastric cancer.

## Introduction

Gastric cancer (GC) has a high morbidity and mortality rate worldwide. Although GC patients can benefit from surgery and chemoradiotherapy, the 5 years survival rate is only 40% (Noh et al., 2014; Bray et al., 2018). The poor survival rate of patients with GC after surgery is mostly from metastasis and recurrence after surgery. Therefore, better understanding of the mechanisms underlying the invasion and metastasis of GC is critical to identify effective treatment targets for GC.

Spindle and kinetochore related complex subunit 3 (SKA3), which is encoded by the SKA3 gene on chromosome 13q12.11, is a newly discovered important factor involved in the formation of the spindle and kinetochore related complex (SKA), which controls mitosis together with the NDC80 complex (Ciferri et al., 2008; Gaitanos et al., 2009; Jeyaprakash et al., 2012; Foley and Kapoor, 2013; Zhang et al., 2017). Recent studies showed that SKA3 plays a cancer-promoting role, not only by affecting the proliferation of tumor cells through regulating cell cycle checkpoints, but also by participating in a variety of cell biological functions to affect the occurrence and development of tumors (Jiao et al., 2013; Pesson et al., 2014; Lee et al., 2015; Hou et al., 2019). However, the role of SKA3 in GC has not been studied.

Dual-specific phosphatase 2 (DUSP2) is a member of the tyrosine-specific phosphatase family (Caunt and Keyse, 2013; Low and Zhang, 2016), which contains more than 30 DUSP proteins. DUSP2 is an important regulator of immune responses, and its low expression has been detected in various tumors, such as prostate cancer, glioma, and leukemia (Hartmann et al., 2016; Dan et al., 2020; Haag et al., 2016). DUSP2 participates in the development of tumor through multiple signaling pathways and negatively regulates the activity of extracellular regulatory protein kinase (ERK) and p38 *in vitro* (Chu et al., 1996; Griffin et al., 2012; Lu et al., 2015). However, the role of DUSP2 in GC has not been clarified.

Here we studied the expression and progression of SKA3 in GC *in vitro* and vivo. SKA3 may be the initiating factor to promote the progression of gastric cancer. The amplification of SKA3 through its downstream cascade signal transduction may be an important reason for the progress of GC. SKA3 may mediate EMT and promote the progression of GC through the DUSP2-ERK1/2 axis. Our results identified the SKA3-DUSP2-ERK1/2 axis as a potential therapeutic target for GC.

## Materials and Methods

### Patient Tissue Specimens

Our study included two independent experimental cohorts. The first group included 35 GC cases with fresh frozen tumors and adjacent tissues selected from January to March 2018 in the Second Hospital of Jilin University, these samples were analyzed by quantitative real-time PCR (qRT-PCR) and western blot analysis. The second group included 164 GC tissue samples from patients who received radical gastrectomy at the Gastrointestinal Cancer Surgery Department of the First Affiliated Hospital of China Medical University from March 2009 to June 2012; these samples were analyzed by immunohistochemistry for SKA3 and DUSP2. All included patients had a single lesion as confirmed by pathological verification and complete postoperative follow-up data; the patients did not receive chemoradiotherapy before surgery. The study protocol was approved by the hospital ethics committee, and all patients signed informed consent.

### Immunohistochemistry

After dewaxing and hydration, paraffin-embedded sections were immersed in 3% H_2_O_2_ solution for 20 min at 37°C to eliminate endogenous peroxidase followed by incubation in sodium citrate buffer (pH 6.0) at 121°C for 2 min. Sections were then incubated with primary antibodies against SKA3 (1:200, Abcam, Cambridge, MA, United States), or DUSP2 (1:200, Abcam) at 4°C overnight. Sections were then incubated with secondary antibodies at 37°C for 30 min and then stained with 3, 3′-diaminobenzidine, followed by counter-staining with hematoxylin for 1 min. Sections were dehydrated in a gradient ethanol series followed by xylene. Samples were scored according to the staining intensity and proportion of positive cells. Staining intensity was scored as follows: 0 for colorless, 1 for yellow, 2 for brown, and 3 for dark brown. The proportion of positive cells was scored as follows: < 5% was 0, 5–25% was 1, 25–50% was 2, 50–75% was 3, and >75% was 4. The final score was the product of the staining intensity and proportion of positive cell scores; samples with scores ≥5 were considered positive and samples with scores ≤4 were negative. Two authors evaluated and scored, and were blinded to the patient’s pathological information. The controversial cases were re-scored and finally reached an agreement.

### Cell Culture and Infection

The immortalized gastric epithelium cell line GES-1 and GC cell lines SGC-7901, MGC-803, HGC-27, and MKN-45 were purchased from the Cell Bank of the Chinese Academy of Sciences (Shanghai, China). Cells were cultured in DMEM or RPMI 1640 medium containing 10% serum. The human peritoneal mesothelial cell (HPMC) HMrSV5 cell line was provided by Youming Peng (Second Hospital of Zhong nan University, Changsha, China) and cultured in RPMI 1640 medium containing 10% serum. Cells were cultured in an incubator at 37°C in 5% CO_2_.

SKA3 silencing lentiviruses (sh-SKA3), DUSP2 silencing lentiviruses (sh-DUSP2), DUSP2 overexpressing lentiviruses (oe-DUSP2) and empty vector virus (control) were produced by Genechem (Shanghai, China). GC cells were seeded in 12-well plates and when cells achieved 60–70% confluence, an appropriate amount of virus was added according to the cell line MOI value. Stably infected cell lines were selected in 5 μg/ml puromycin or neomycin. The sequences used to contrast sh-SKA3 are follows: sh-1, CTT​ATG​AGA​ATC​TGC​TCA​GAA and sh-2, GCA​TAG​CTT​TGG​TAT​CCA​CAA. The sequence used for sh-DUSP2 is CAT​CTG​TCT​GGC​ATA​CCT​CAT and the control sequence is TTC​TCC​GAA​CGT​GTC​ACG​T.

### qRT-PCR

Total RNA was extracted from GC tissues and cells by Trizol reagent (Invitrogen, Carlsbad, CA, United States). cDNA was generated by reverse transcription reaction using a reverse transcription kit (Takara, Tokyo, Japan), and the SYBR Premix Ex Taq ™ kit (Takara, Japan) was used to conduct qRT-PCR. The experiment was repeated three times. GAPDH mRNA was used as the internal reference gene. Data expression was determined by the −ΔΔCt method (Livak and Schmittgen, 2001).

The primer sequences for qRT-PCR are as follows.

SKA3 forward: 5′-GCC​ATA​GAT​ACA​GAA​TCC​AGG​CT-3′

SKA3 reverse: 5′-CCA​AAG​GAG​AGT​TGG​TAT​ATT​CGG-3′

DUSP2 forward: 5′-TGT​GGA​GGA​CAA​CCA​GAT​GGT​G-3′

DUSP2 reverse: 5′-GAG​GTA​TGC​CAG​ACA​GAT​GGT​G-3′

GAPDH forward: 5′-GTC​TCC​TCT​GAC​TTC​AAC​AGC​G-3′

GAPDH reverse: 5′-ACC​ACC​CTG​TTG​CTG​TAG​CCA​A-3′

#### Western Blot

Total protein was extracted from tissues and cells using lysis buffer. Samples were separated by SDS-PAGE and transferred to a PVDF membrane. The membrane was blocked in a 5% skim milk for 1 h. The membrane was then incubated overnight at 4°C with primary antibody, followed by incubation in secondary antibody for 1 h. ECL luminescent solution was used to visualize protein bands. Antibodies against SKA3 (ab118560), DUSP2 (ab137640), and GAPDH (ab181602) were purchased from Abcam and antibodies against ZEB1 (#70512), E-cadherin (#14472), N-cadherin (#13116), Vimentin (#5741) Erk1/2 (#4695), and p-Erk1/2 (#4370) were obtained from Cell Signaling Technology, antibodies against PCNA (10205-2-AP), c-MYC (67447-1-Ig), Cyclin D1 (26939-1-AP) were obtained from proteintech group.

### Co-Immunoprecipitation

HGC-27 cell lysates were incubated with primary antibody (or IgG as control) and A/G agarose beads at 4°C for 6 h. After three washes with IP buffer, samples were analyzed by western blot.

### Cell Proliferation and Colony Formation Assays

Cell viability was measured using the Cell Counting Kit-8 (CCK8, MedChemExpress, United States). GC cells were seeded at 2 × 10^3^ cells/well in 96-well plates. After culture for 24, 48, 72 and 96 h, 10 μl CCK8 solution was added to each well. After incubation for 2 h, the absorbance value was measured at 450 nm using a Biotek microplate reader.

For colony formation assays, cells were seeded in 6-well plates (1 × 10^3^ cells/well). After culture for 14 days, the cells were fixed with paraformaldehyde for 20 min and stained with 0.4% Trypan blue for 15 min for clone counting.

### Flow Cytometry

Cells collected by trypsinization were fixed overnight with 900 μl of pre-cooled 75% ethanol at 4°C. Cells were rinsed with PBS and incubated in 300 μl propidium iodide staining solution at 37°C for 30 min in the dark. Cell cycles distribution was then analyzed by flow cytometry.

### Wound-Healing, Cell Migration and Invasion Assays

For wound-healing experiments, cells were uniformly seeded in a 6-well plate. Once cells achieved 80% confluence, a 100 μl pipette tip was used to scratch the cell monolayer. The same field of vision was photographed under an inverted microscope at 0 and 48 h after the scratch, and the scratch width was measured.

For migration experiments, a Transwell chamber (8 μm, Corning, United States) was placed in a 24-well plate and cells were seeded into the upper chamber at a density of 2 × 10^4^/well. Serum-free medium (100 μl) was added to the top well, and the lower chamber was filled with 600 μl medium containing 10% serum. After 16 h of culture, the Transwell chamber was removed, fixed with paraformaldehyde, and stained with 0.4% trypan blue.

For invasion experiments, 90 μl of diluted Matrigel (BD Biosciences, NJ, United States) was added to the upper chamber and 2 × 10^4^/well cells were also seeded into the upper chamber. The subsequent steps were the same as for the migration experiment.

### Adhesion Assay

HMrSV5 cells were seeded in a 12-well plate 1 day in advance and GC cells were seeded in a 6 cm Petri dish. Next, 2 μl Calcein-AM (Sigma, United States) was added to GC cells and cells were incubated in the dark for 1 h. The stained GC cells (1 ×10^4^) were then added to each well of the plated HMrSV5 cells. The cells were incubated in the dark for 1 h and then washed. The number of GC cells that adhered to HMrSV5 cells was counted under a fluorescence microscope.

### Animal Experiments

All animal experiments were approved by the Ethics Committee of China Medical University and all operations were in accordance with animal ethic guidelines.

A subcutaneous tumor model was generated in 12 BALB/c immunodeficient nude mice (SPF level; 4–5 weeks old, female, and approximately 20 g in weight). The MGC-803 cells infected with lentivirus for SKA3 silencing or controls were injected into 3 × 10^6^ cells respectively in the left and right axillae of nude mice. Tumor volume was measured every 3 days. After 4 weeks, the subcutaneous tumors were dissected and weighed.

An intraperitoneal metastasis model was established in 20 BALB/c nude mice. Mice were assigned to two groups (*n* = 10 per group), and 5 × 10^6^ MGC-803 cells infected with lentivirus for SKA3 silencing or controls were injected into the abdominal cavity. After 6 weeks, mice were killed and dissected. The number of tumors on the peritoneal tissue was observed.

RNA-seq analysisTrizol reagent was used to extract the total RNA of the MGC-803 silencing and control groups of cells, and each group of cells repeated three biological samples. The subsequent RNA-seq analysis was completed by professional sequencing company. The sequencing data is not uploaded to the GEO public database, but owned by ourselves.

### Statistical Analysis

Statistical analysis was performed using SPSS 22.0 and GraphPad Prism 7.0. Experiments were repeated three times. Data are expressed as mean ± SD. The unpaired *t* test was used to compare the groups. The chi-square test was used to analyze the relationship between SKA3 expression and clinicopathological factors. The Pearson test was used to analyze the correlation between SKA3 and DUSP2 expression. Survival analysis was performed using the Kaplan–Meier method. Log-rank test calculated the survival differences among different groups and fitted the univariate and multivariate Cox proportional hazards model. *p* < 0.05 was considered statistically significant.

## Results

SKA3 is highly expressed in GC, negatively correlates with DUSP2 expression and is associated with poor prognosis in GC.

**FIGURE 1:**
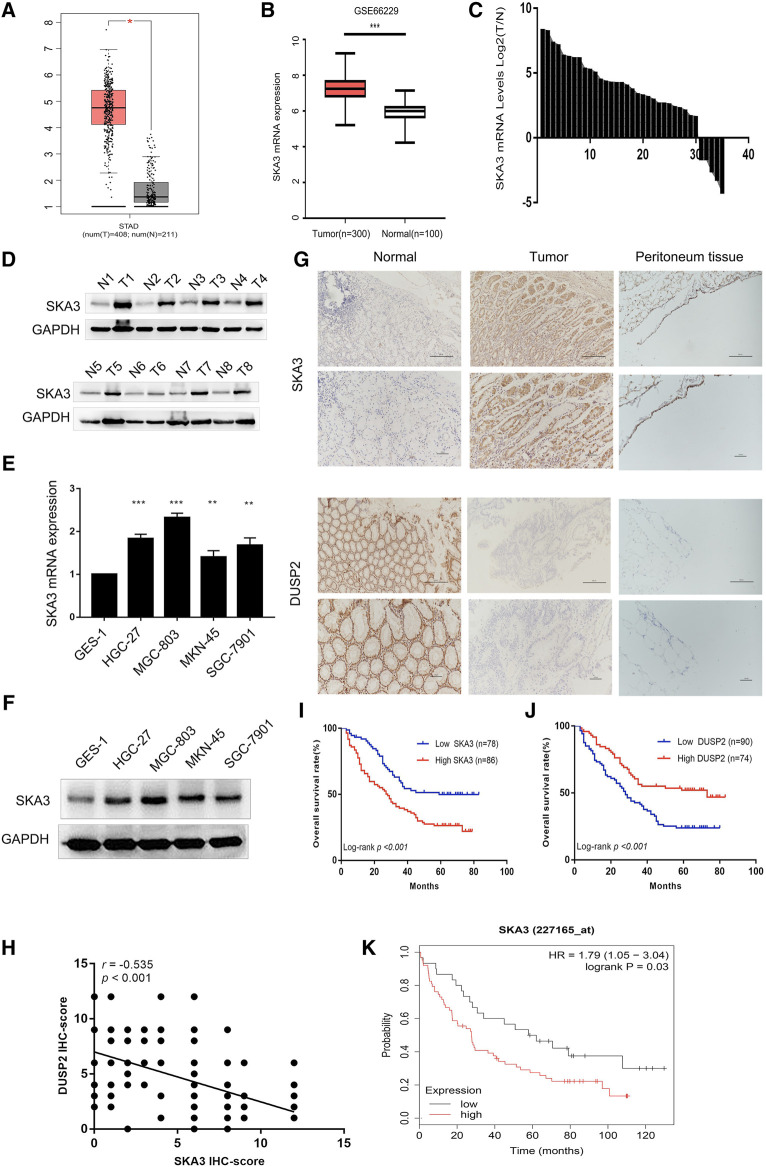
SKA3 is upregulated in gastric cancer. **(A)** SKA3 mRNA expression in TCGA database. **(B)** SKA3 mRNA expression in GEO database. **(C)** SKA3 mRNA expression in gastric cancer tissues. **(D)** SKA3 protein levels in gastric cancer tissues. N, normal tissue. T, tumor tissue. **(E)** SKA3 mRNA expression in gastric cancer cell lines. **(F)** SKA3 protein levels in gastric cancer cell lines. **(G)** SKA3 and DUSP2 expression in gastric cancer tissues, normal tissues, peritoneal tissues by immunohistochemistry (IHC). **(H)** The correlation between SKA3 and DUSP2 IHC score by scatter plots. **(I)** Kaplan-Meier survival curves about SKA3. **(J)** Kaplan-Meier survival curves about DUSP2. **(K)** The prognostic significance of SKA3 high and low expression in Kaplan-Meier plotter database.*p < 0.05, **p < 0.01, ***p < 0.001.

We analyzed the expression level of SKA3 mRNA in GC in TCGA (Figure 1A) and GEO databases (GSE66229) (Figure 1B) and found that SKA3 was highly expressed in GC in both databases. Analysis of 35 GC samples and adjacent tissues by qRT-PCR (Figure 1C) and western blot (Figure 1D) showed that SKA3 mRNA and protein expression was up-regulated in GC samples. We also examined SKA3 mRNA (Figure 1E) and protein expression (Figure 1F) in the GC cell lines SGC-7901, MGC-803, HGC-27 and MKN-45 along with GES-1 cells by qRT-PCR and western blot. SKA3 mRNA and protein was highly expressed in GC cell lines but not in GES-1 cells.

We next performed immunohistochemistry of 164 GC tumor samples and found that SKA3 and DUSP2 were mainly expressed in the cytoplasm and nucleus (Figure 1G). The positive expression rates of SKA3 and DUSP2 were 52.44% (86/164) and 45.12% (74/164), respectively. There was a negative correlation between SKA3 expression and DUSP2 expression in GC (Pearson test, *r* = −0.535; *p* < 0.001, Figure 1H).

High SKA3 expression was significantly correlated with Borrmann type, N stage, and peritoneal metastasis (all *p* < 0.05, Table 1). Kaplan–Meier analysis showed that patients with high expression of SKA3 (Figure 1I) and low expression of DUSP2 (Figure 1J) showed a worse overall survival (OS) compared with low expression of DUSP2. Kaplan–Meier analysis also showed that high expression of SKA3 correlated with worse OS (Figure 1K). Cox multivariate analysis further confirmed that Borrmann type, T stage, N stage, peritoneal metastasis, and SKA3 high expression were independent prognostic factors for OS (HR = 1.829, 95% CI: 1.187–2.818, *p* = 0.005; HR = 1.950, 95% CI: 1.028–3.702, *p* = 0.028; HR = 2.768, 95% CI: 1.471–5.209, *p* = 0.001; HR = 2.210, 95%CI: 1.125–4.342, *p* = 0.021; HR = 1.542, 95% CI: 1.003–2.372, *p* = 0.049, respectively) (Table 2).

**TABLE 1:** Relationship between the SKA3 expression and clinical parameter.

Variables	SKA3 expression		*p*
	High (*n* = 86) Low (*n* = 78)		
Age			
<60	49	34	0.087
≥60	37	44	
Sex			
Male	55	53	0.059
Female	31	25	
Size			
<5 (cm)	21	30	0.052
≥5 (cm)	65	48	
Differentiation			
Well	8	7	0.753
Moderate	31	24	
Poor	47	47	
Tumor location			
Upper	11	9	0.088
Middle	18	7	
Lower	57	62	
Borrmann type			
EGC + I–III	38	72	<0.001
Ⅳ	48	6	
Invasive depth			
T1-T2	12	16	0.265
T3-T4	74	62	
lymph node metastasis			
N0	14	25	0.018
N1-N3	72	53	
Peritoneal metastasis (M)			
M0	73	76	0.005
M1	13	2	
Lymph vessel tumoremboli			
Absent	58	48	0.430
Present	28	30	
Stage (TNM)			
Ⅰ	3	7	0.491
Ⅱ	20	17	
Ⅲ	54	48	
Ⅳ	9	6	

Abbreviations: SKA3, Spindle and kinetochore-related complex subunit 3; EGC, Early gastric cancer.

**TABLE 2:** The univariate and multivariate analyses of factors associated with overall survival.

Variable	Univariate cox regression		Multivariate cox regression	
	HR (95% CI)	*p*-value	HR (95% CI)	*p*-value
Age (<60)	1.028 (0.696–1.519)	0.888		
Sex (male)	1.224 (0.816–1.835)	0.328		
Size (>5cm)	1.937 (1.223–3.067)	0.005		
Differentiation (Poor)	1.011 (0.681–1.503)	0.956		
Tumor location (Middle, low)	0.983 (0.456–2.120)	0.983		
Borrmann type (Ⅳ)	2.455 (1.643–3.669)	<0.001	1.829 (1.187–2.818)	0.005
Invasive depth (T3-T4)	2.607 (1.390–4.889)	0.003	1.950 (1.028–3.702)	0.028
lymph node metastasis (+)	3.204 (1.709–6.006)	<0.001	2.768 (1.471–5.209)	0.001
Peritoneal recurrence (+)	2.536 (1.310–4.912)	0.006	2.210 (1.125–4.342)	0.021
Lymph vessel tumoremboli (+)	1.126 (0.749–1.693)	0.568		
High SKA3 expression	1.926 (1.284–2.890)	0.002	1.542 (1.003–2.372)	0.049

Abbreviations: SKA3, Spindle and kinetochore-related complex subunit 3.

SKA3 promotes proliferation, migration, invasion and adhesion of GC cells.

**FIGURE 2:**
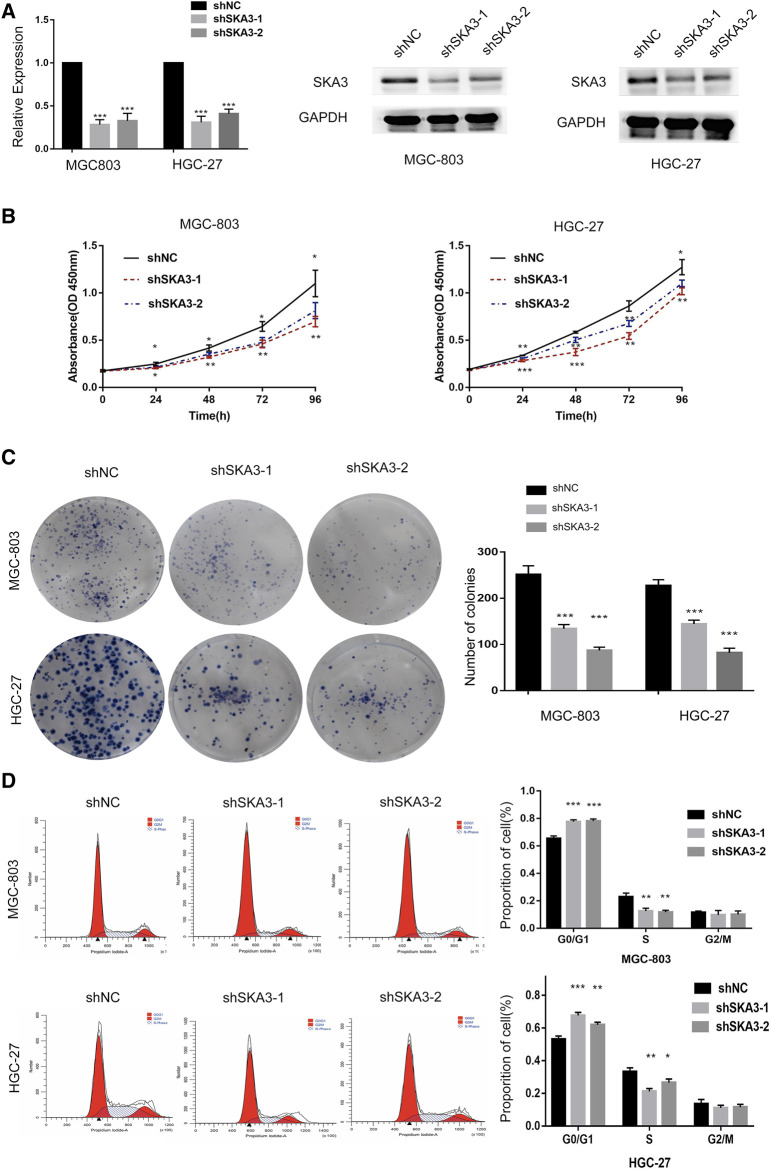
SKA3 promotes proliferation in gastric cancer. **(A)** mRNA and protein levels of SKA3 after transfection with shSKA3 lentivirus in MGC-803 and HGC-27. **(B)** CCK-8 cell proliferation assay detects cell viability by silenced for SKA3 in MGC-803 and HGC-27. **(C)** Cloning experiment detects cell growth by silenced for SKA3 in MGC-803 and HGC-27. **(D)** Flow cytometry detects cell cycle n by silenced for SKA3 in MGC-803 and HGC-27. Data are shown as mean ± SD for triplicate experiments. The two-tailed Student’s unpaired *t*-test was used. *p < 0.05, **p < 0.01, ***p < 0.001.

Our results showed that high SKA3 expression was detected in MGC-803 and HGC-27 cells. Therefore, these cell lines were selected to generate cells with lentivirus-mediated stable knock-down of SKA3 (MGC-803-shSKA3 and HGC-27-shSKA3). Cells infected with lentivirus expressing control were designated as shNC. qRT-PCR and western blot were used to confirm knock-down efficiency (Figure 2A).

**FIGURE 3:**
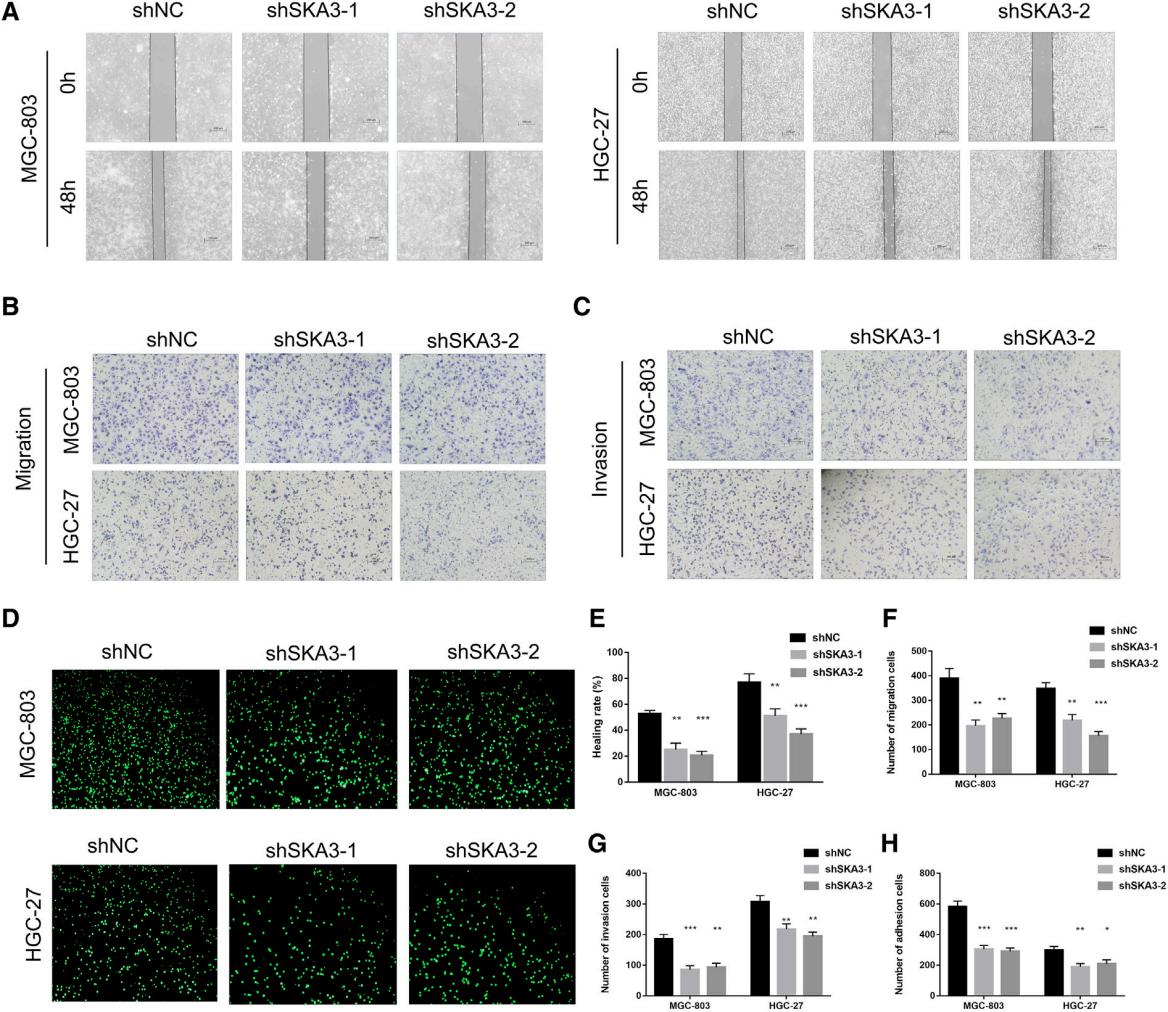
SKA3 promotes migration, invasion and adhesion in gastric cancer. **(A)** Wound-healing experiments detects cell migration by silenced for SKA3 in MGC-803 and HGC-27. **(B)** Transwell experiment detects cell migration by silenced for SKA3 in MGC-803 and HGC-27. **(C)** Transwell experiment detects cell invasion by silenced for SKA3 in MGC-803 and HGC-27. **(D)** Adhesion experiment detects cell adhesion by silenced for SKA3 in MGC-803 and HGC-27. **(E)** Relative healing rate in **(A)** were quantified and shown. **(F)** Relative migration numbers in **(B)** were quantified and shown. **(G)** Relative invasion numbers in **(C)** were quantified and shown. **(H)** Relative adhesion numbers in **(D)** were quantified and shown. Data are shown as mean ± SD for triplicate experiments. The two-tailed Student’s unpaired *t*-test was used. **p < 0.01, ***p < 0.001.

CCK-8 cell proliferation assay showed that the viability of cells silenced for SKA3 was significantly lower than that of the control cells (Figure 2B). Colony formation assays showed that the number of cell clones in SKA3-silenced cells was less than that in the control cells (*p* < 0.05) (Figure 2C). Flow cytometry revealed that cells silenced for SKA3 exhibited a cell cycle arrest in G0/G1 phase (Figure 2D). Wound-healing experiments showed that silencing SKA3 expression inhibited cell migration (Figures 3A,E), and Transwell migration and invasion experiments showed that silencing SKA3 expression also significantly inhibited the migration and invasion of GC cells (Figures 3B,C,F,G). Our results showed that high expression of SKA3 was associated with peritoneal metastasis, and we thus carried out adhesion experiments. The number of GC cells silenced for SKA3 that adhered to HPMCs was significantly lower than that in the control group (Figures 3D,H). These results indicated that reduced expression of SKA3 resulted in inhibited proliferation and adhesion ability of GC cells and made cells less prone to peritoneal metastasis.

SKA3 regulates epithelial-mesenchymal transition (EMT) through the MAPK/ERK pathway.

**FIGURE 4:**
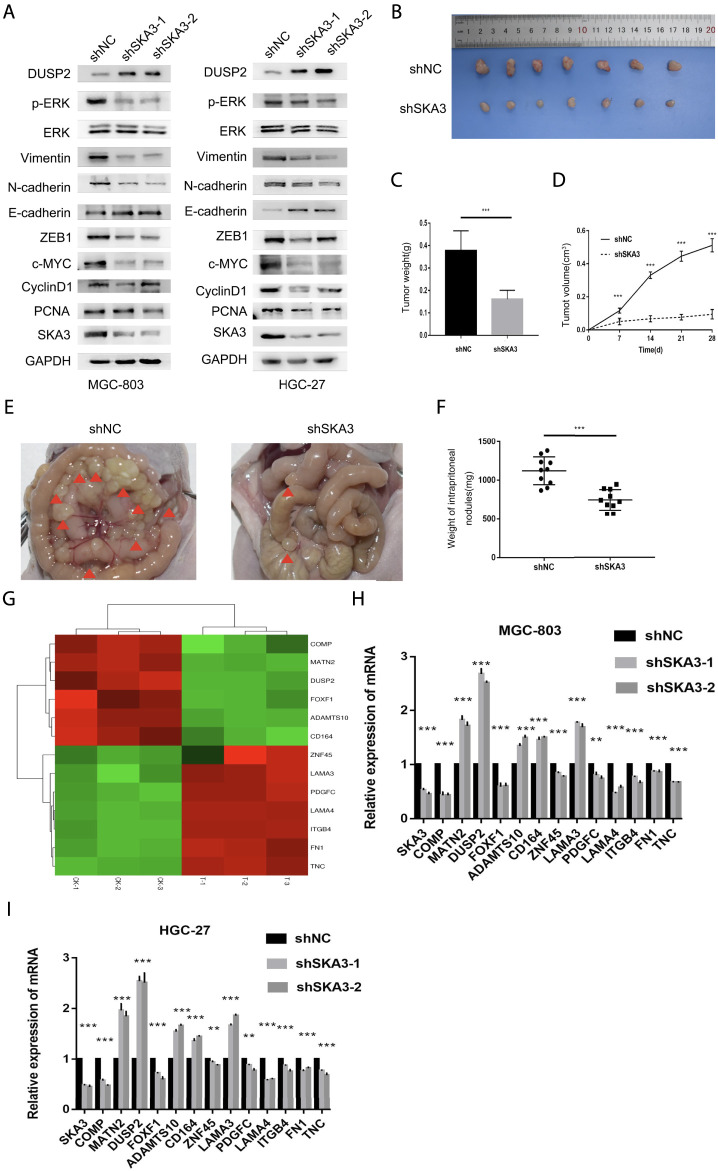
SKA3 regulates epithelial-mesenchymal transition (EMT), MAPK/ERK pathway *in vitro* and regulates the aggressive behaviors *in vivo*. **(A)** Silenced for SKA3 inhibits proliferation, EMT and MAPK/ERK related proteins in MGC-803 and HGC-27. **(B)** Tumor growth assessment in subcutaneous tumor model of gastric cancer. **(C)** Tumor weight was measured in shSKA3 and control groups. **(D)** Tumor volume was measured in shSKA3 and control groups. **(E)** Intraperitoneal metastasis model showed that the metastatic nodules derived from shSKA3 and control groups. **(F)** Intraperitoneal metastasis tumor weight was measured in shSKA3 and control groups. **(G)** Microarray heatmap showed differentially expressed genes. **(H)** Differentially expressed genes were screened by qRT-PCR in MGC-803. **(I)** Differentially expressed genes were screened by qRT-PCR in HGC-27. **(J)** Western blot of DUSP2 levels in the sh-SKA3 cells. Data are shown as mean ± SD for triplicate experiments. The two-tailed Student’s unpaired *t*-test was used. *p < 0.05, **p < 0.01, ***p < 0.001.

Since abnormal activation of EMT is an important biological process in tumor progression, we examined the EMT-related markers ZEB1, E-cadherin, N-cadherin and Vimentin in GC cells. Western blot showed that silencing SKA3 n GC cells resulted in the up-regulation of E-cadherin and down-regulation of ZEB1, N-cadherin, and Vimentin. The level of phosphorylated ERK1/2 (Thr202/Tyr204) was also decreased in SKA3 silenced cells, while there was no change in overall ERK1/2 level. Proliferation-related markers, PCNA, c-MYC and Cyclin D1 were also decreased in SKA3 silenced cells. These results suggest that silenced SKA3 inhibited proliferation, also through ZEB1 inhibited EMT and the MAPK/ERK pathway (Figure 4A).

### SKA3 Promotes GC Proliferation and Metastasis *in vivo*

We next established a subcutaneous tumor model and examined the effects of SKA3 knockdown on tumor growth (Figure 4B). We found that tumor growth was reduced in the SKA3 silenced group; the volume and weights of tumors derived from SKA3 silenced cells were smaller than those in the control group (Figures 4C,D).

We further studied the impact of SKA3 silencing on the metastatic ability of GC *in vivo* using an intraperitoneal metastasis model (Figure 4E). While extensive and diffuse cancer nodules in the abdominal cavity were observed in the control group, the weight of nodules in the SKA3 silenced group was significantly reduced (Figure 4F). Together, these results showed that reduced expression of SKA3 inhibited the proliferation, invasion and metastasis of GC *in vivo*.

DUSP2 inhibits the MAPK/ERK pathway activity through binding ERK1/2 and inhibits the proliferation, migration, invasion, adhesion and EMT of GC cells.

To study the mechanism by which SKA3 promotes the progression of GC, we performed RNA-seq using MGC-803 cells silenced for SKA3 compared with controls. The results revealed 997 genes that were upregulated and 236 genes that were downregulated in MGC-803 cells silenced for SKA3. We screened out the downstream gene DUSP2 (Figure 4G). Previous studies showed that DUSP2 functions as an upstream negative regulator of the MAPK/ERK pathway. The MAPK/ERK pathway plays an important role in the progression of GC (Wang et al., 2012; Hu J. et al., 2018). We screened DUSP2 as a key downstream gene through qRT-PCR and western blot (Figures 4H–I).

**FIGURE 5:**
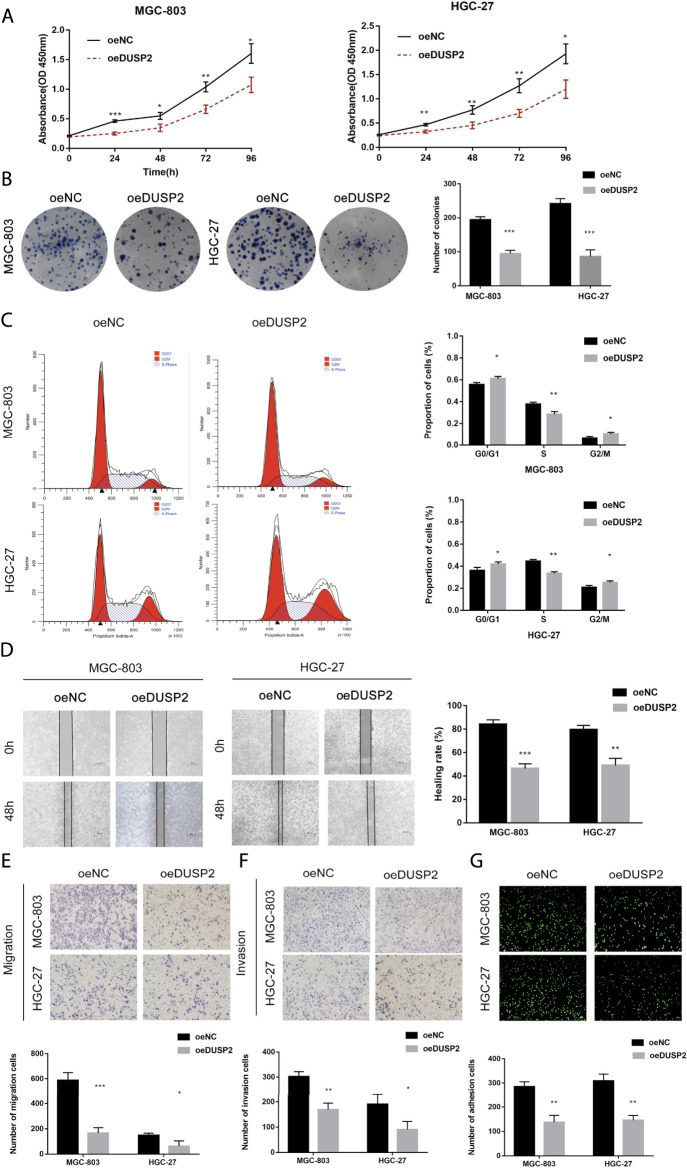
DUSP2 inhibits proliferation, migration, invasion and adhesion in gastric cancer. **(A)** CCK-8 cell proliferation assay detects cell viability by overexpression for DUSP2 in MGC-803 and HGC-27. **(B)** Cloning experiment detects cell growth by overexpression for DUSP2 in MGC-803 and HGC-27. **(C)** Flow cytometry detects cell cycle by overexpression for DUSP2 in MGC-803 and HGC-27. **(D)** Wound-healing experiments detects cell migration by overexpression for DUSP2 in MGC-803 and HGC-27. **(E)** Transwell experiment detects cell migration by overexpression for SKA3 in MGC-803 and HGC-27. **(F)** Transwell experiment detects cell invasion by overexpression for SKA3 in MGC-803 and HGC-27. **(G)** Adhesion experiment detects cell adhesion by overexpression for SKA3 in MGC-803 and HGC-27. Data are shown as mean ± SD for triplicate experiments. The two-tailed Student’s unpaired *t*-test was used. *p < 0.05, **p < 0.01, ***p < 0.001.

**FIGURE 6:**
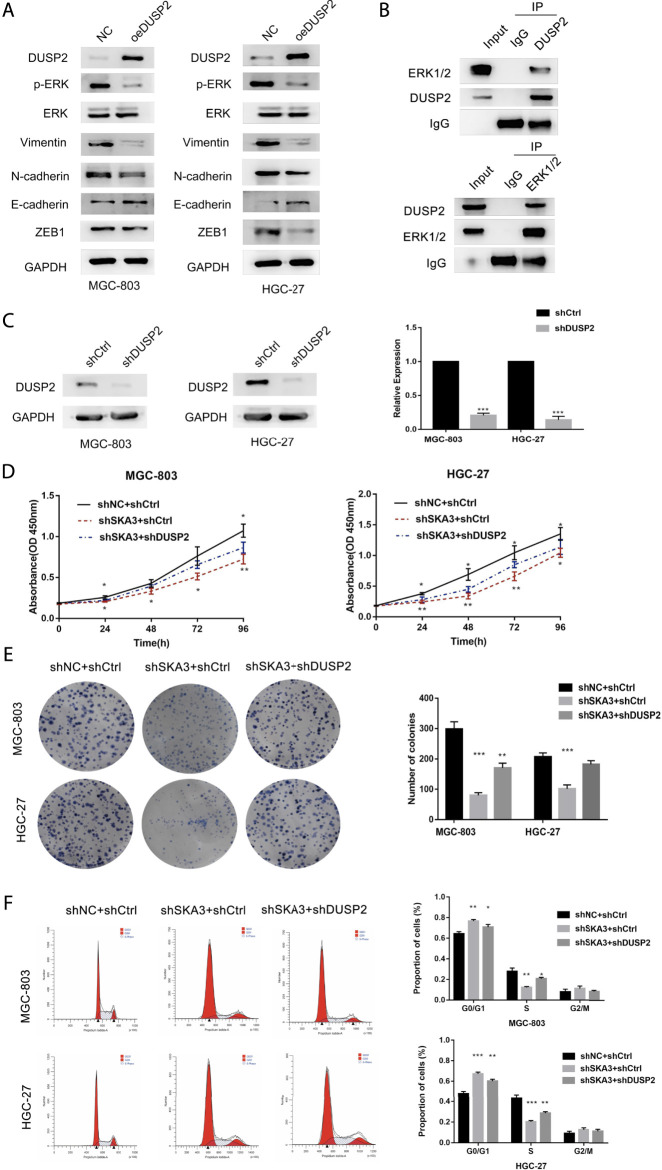
SKA3 mediates DUSP2 induced proliferation, EMT by inactivating MAPK/ERK pathway. **(A)** Overexpression for DUSP2 inhibits EMT and MAPK/ERK related proteins in MGC-803 and HGC-27. **(B)** HGC-27 cell total lysates were IP with the DUSP2 antibodies or ERK1/2 antibodies, followed by Western blotting using the indicated antibodies. **(C)**. mRNA and protein levels of DUSP2 after transfection with shDUSP2 lentivirus in MGC-803 and HGC-27. **(D)** The inhibiting effect of SKA3 downregulation on MGC-803 and HGC-27 CCK-8 cell proliferation was rescued by shDUSP2 transfection. **(E)** The inhibiting effect of SKA3 downregulation on MGC-803 and HGC-27 CCK-8 cell cloning was rescued by shDUSP2 transfection. **(F)** The inhibiting effect of SKA3 downregulation on MGC-803 and HGC-27 cell cycle was rescued by shDUSP2 transfection. Data are shown as mean ± SD for triplicate experiments. The two-tailed Student’s unpaired *t*-test was used. *p < 0.05, **p < 0.01, ***p < 0.001.

We established two stable cell lines overexpressing DUSP2 (MGC-803-oe-DUSP2 and HGC-27-oe-DUSP2), as well as a control (oeNC). Western blot was used to confirm overexpression efficiency in the DUSP2 overexpressing cell lines (Figure 6A). CCK-8 cell proliferation assays showed that the cell proliferation ability of the DUSP2 overexpression group was significantly lower than that of the control group (Figure 5A). Colony formation assays showed that the number of cell clones in the DUSP2 overexpression group was less than that in the control group (*p* < 0.05) (Figure 5B). Cell cycle analysis of the DUSP2 overexpression group showed a cell cycle arrest in G0/G1 and G2/M phase (Figure 5C). Wound-healing experiments showed that DUSP2 overexpression inhibited the migration of GC cells (Figure 5D), and Transwell migration and invasion experiments showed that DUSP2 overexpression also significantly inhibited the migration and invasion of GC cells (Figures 5E,F). In adhesion experiments, we found that the number of GC cells adhering to mesothelial cells in the DUSP2 overexpressed group was significantly lower than that in the control group, indicating that overexpression of DUSP2 inhibited the adhesion ability of GC cells (Figure 5G). Western blot showed that DUSP2 overexpression resulted in up-regulation of E-cadherin and down-regulation of ZEB1, N-cadherin, and Vimentin (Figure 6A). We performed co-immunoprecipitation experiments and found that DUSP2 binds to ERK1/2 (Figure 6B), thereby dephosphorylating ERK1/2 and inhibiting MAPK/ERK pathway activity.

Knockdown of DUSP2 partially rescued the effects on proliferation and invasion caused by SKA3 knockdown.

**FIGURE 7:**
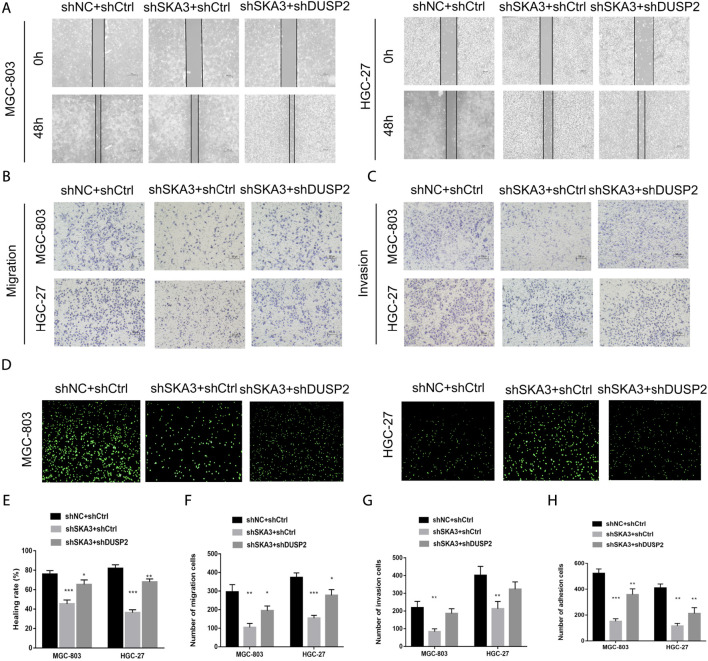
SKA3 mediates DUSP2 induced migration, invasion and adhesion by inactivating MAPK/ERK pathway. **(A)** The inhibiting effect of SKA3 downregulation on MGC-803 and HGC-27 wound-healing was rescued by shDUSP2 transfection. **(B)** The inhibiting effect of SKA3 downregulation on MGC-803 and HGC-27 migration was rescued by shDUSP2 transfection. **(C)** The inhibiting effect of SKA3 downregulation on MGC-803 and HGC-27 invasion was rescued by shDUSP2 transfection. **(D)** The inhibiting effect of SKA3 downregulation on MGC-803 and HGC-27 adhesion was rescued by shDUSP2 transfection. **(E)** Relative healing rate in **(A)** were quantified and shown. **(F)** Relative migration numbers in **(B)** were quantified and shown. **(G)** Relative invasion numbers in **(C)** were quantified and shown. **(H)** Relative adhesion numbers in **(D)** were quantified and shown. Data are shown as mean ± SD for triplicate experiments. The two-tailed Student’s unpaired *t*-test was used. *p < 0.05, **p < 0.01, ***p < 0.001.

**FIGURE 8:**
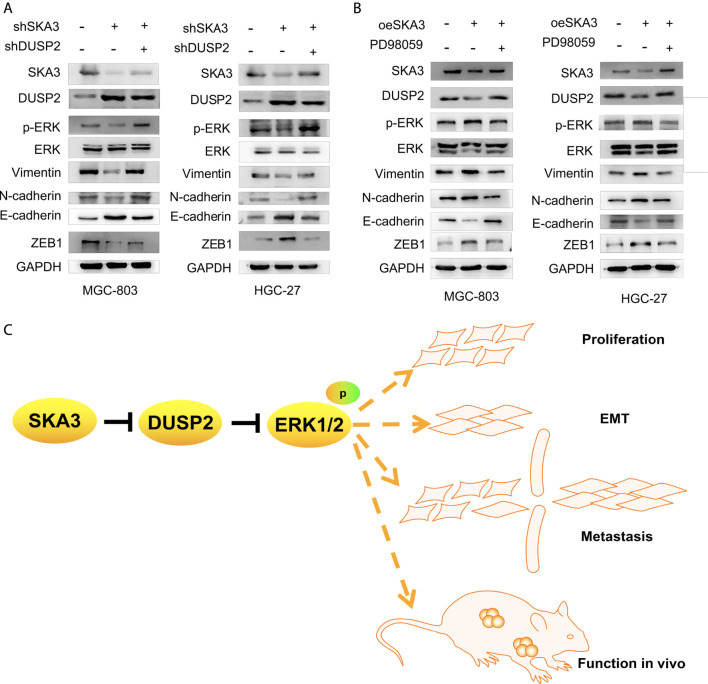
A model illustrating SKA3-DUSP2-ERK1/2 axis in gastric cancer. **(A)** Co-transfected with shSKA3 and shDUSP2 rescue EMT and MAPK/ERK related proteins compared with shSKA3 group in MGC-803 and HGC-27. **(B)** Co-transfected with oeSKA3 and PD98059 rescue EMT and MAPK/ERK related proteins compared with oeSKA3 group in MGC-803 and HGC-27. **(C)** Model of SKA3-DUSP2-ERK1/2 axis in gastric cancer.

To verify the SKA3-DUSP2-ERK1/2 signal axis, we performed functional rescue experiments by co-infecting GC cells with shSKA3 and shDUSP2. First, we verified the DUSP2 silencing efficiency in co-infected cells (Figure 6C). The co-infected group showed partially increased cell proliferation (Figures 6D,E), G0/G1 phase population (Figure 6F), wound-healing ability (Figures 7A,E), migration (Figures 7B,F), invasion (Figures 7C,G) and adhesion (Figures 7D,H) compared with SKA3 knockdown cells. Western blot showed that SKA3, ZEB1, N-cadherin, Vimentin, and phosphorylated ERK1/2 expression were partially increased, E-cadherin expression was partially decreased, and total ERK1/2 was unchanged in the double knockdown cells compared with SKA3 knockdown cells (Figure 8A). We also used the ERK1/2 pathway inhibitor PD98059 in cells with overexpression of SKA3, and western blot showed that SKA3, ZEB1, N-cadherin, Vimentin, and phosphorylated ERK1/2 expression were partially decreased, E-cadherin expression was partially increased, and total ERK1/2 was unchanged compared with cells with SKA3 overexpression (Figure 8B). These results show that the effect of SAK3 on proliferation and metastasis is likely largely achieved through downregulation of DUSP2. Our findings suggest that SKA3 negatively regulates DUSP2, through binding ERK1/2 to activate the MAPK/ERK pathway to regulate the malignant phenotype of GC (Figure 8C).

## Discussion

The progression of GC is the result of the joint action of a variety of factors. The progression from the initiation of cancer to invasion and metastasis takes a certain amount of time. Tumor invasion and metastasis are extremely complex dynamic processes and the most essential feature of malignant tumors. Therefore, research in GC has focus on exploring the mechanism of invasion and metastasis with the aim to identify potential targets (Wadhwa et al., 2013; Hayakawa et al., 2015; Parker et al., 2016; Van cutsem et al., 2016; Yang et al., 2019).

SKA3 is highly expressed in malignant tumors such as colon cancer, liver cancer, cervical cancer, and prostate cancer and plays an important role in cancer occurrence and development (Lee et al., 2015; Chuang et al., 2016; Hu R. et al., 2018; Hou et al., 2019). However, the expression of SKA3 and its role in tumorigenesis in GC has been unknown. Our study found that high expression of SKA3 in GC tissues was associated with N stage and peritoneal metastasis, and patients with high expression of SKA3 showed worse prognosis. Chuang et al. found that SKA3 regulates the cell cycle, apoptosis, proliferation and invasion in colorectal cancer and that SKA3 expression is associated with poor prognosis (Pastushenko et al., 2018). Hu et al. found that SKA3 promotes cell proliferation and invasion by regulating the PI3K/Akt pathway in cervical cancer (Hu J. et al., 2018). Our study found that SKA3 activates the MAPK/ERK pathway by regulating DUSP2. We also found that SKA3 promotes GC proliferation and EMT as well as GC invasion and even peritoneal metastasis. This is the first study to reveal the relationship between SKA3 and clinicopathological factors and the mechanisms in GC.

EMT is generally regulated by upstream transcription factors. When EMT occurs, cell adhesion ability is reduced and migration ability is improved. EMT therefore plays a key role in cancer progression, as it results in tumor cell detachment from the primary focus and infiltration to distal sites (Moreno-Bueno et al., 2008; Lv et al., 2010; Pastushenko et al., 2018; Dongre and Weinberg, 2019). The MAPK/ERK pathway promotes GC cell migration and invasion through regulating EMT. GC cells mediated by the apoptosis factor FAS ligand can inhibit the phosphorylation of GSK-3β through ERK1/2, thereby reducing the degradation of β-catenin by GSK-3β and promoting the accumulation of β-catenin and SNAIL proteins in the nucleus. EMT is thus induced in GC cells. Both β-catenin and SNAIL protein expressions can be blocked by the ERK inhibitor U0126 (Zheng H. et al., 2013; Zheng HX. et al., 2013).

Our study identified DUSP2 as a downstream key gene of SKA. DUSP2 is expressed at low levels in a variety of tumors, such as prostate cancer, pancreatic cancer, colon cancer, and leukemia, and it participates in the regulation of tumor development through various signaling pathways (Rohan et al., 1993; Hartmann et al., 2016; Hou et al., 2017; Hu R. et al., 2018; Yin et al., 2019). A previous study showed that DUSP2 negatively regulates ERK and p38 activity *in vitro* (Dickinson and Keyse, 2006). Our research confirms that DUSP2 plays a tumor suppressive role in GC and that DUSP2 binds to ERK1/2, thereby promoting its dephosphorylation and inhibiting the MAPK/ERK pathway, resulting in EMT and reduced GC invasion and even peritoneal metastasis. In following the “seed and soil” theory (Paget, 1989), our study indicates that GC cells generate EMT, which makes them prone to metastasis, while DUSP2 is related to hypoxia (Karakashev and Reginato, 2015). Hypoxia changes the microenvironment of the abdominal cavity. Our adhesion experiments showed that mesothelial cells affected by the changes of microenvironment promote the adhesion ability of GC cells. We have identified a new carcinogenic mechanism involving a SKA3-DUSP2-ERK1/2 axis. The mechanism by which SK3 negatively regulates DUSP2 and DUSP2 to inhibit the specific phosphorylation sites of ERK pathway needs to be further explored, which is the limitation of our article.

In conclusion, our study found that SKA3 activates the MAPK/ERK pathway by regulating DUSP2, promoting proliferation and EMT in GC and leading to invasion and peritoneal metastasis. As an independent prognostic factor for GC, SKA3 may be a potential therapeutic target and predictive indicator in GC.

## Data Availability

Publicly available datasets were analyzed in this study. This data can be found here: https://www.ncbi.nlm.nih.gov/geo/query/acc.cgi?acc = GSE66229.
